# Bridging the gap between rectifying developables and tangent developables: a family of developable surfaces associated with a space curve

**DOI:** 10.1098/rspa.2020.0617

**Published:** 2021-02

**Authors:** Brian Seguin, Yi-chao Chen, Eliot Fried

**Affiliations:** ^1^ Department of Mathematics, Loyola University Chicago, Chicago, IL 60660-1537, USA; ^2^ Department of Mechanical Engineering, University of Houston, Houston, TX 77204-4006, USA; ^3^ Mathematics, Mechanics, and Materials Unit, Okinawa Institute of Science and Technology Graduate University, Onna, Okinawa 904-0495, Japan

**Keywords:** ruled surface, rectifying developable, tangent developable, geodesic curvature

## Abstract

There are two familiar constructions of a developable surface from a space curve. The tangent developable is a ruled surface for which the rulings are tangent to the curve at each point and relative to this surface the absolute value of the geodesic curvature *κ*_*g*_ of the curve equals the curvature *κ*. The alternative construction is the rectifying developable. The geodesic curvature of the curve relative to any such surface vanishes. We show that there is a family of developable surfaces that can be generated from a curve, one surface for each function *k* that is defined on the curve and satisfies |*k*| ≤ *κ*, and that the geodesic curvature of the curve relative to each such constructed surface satisfies *κ*_*g*_ = *k*.

## 1. Introduction

The two fundamental notions of curvature for a surface in three space dimensions are the mean and Gaussian curvatures. Surfaces with vanishing mean curvature, called minimal surfaces, have been studied in great detail for both their unique mathematical properties and their remarkable prominence in applications. Surfaces with vanishing Gaussian curvature, called developables, are also of particular interest since they can be smoothly flattened onto a plane without stretching or contraction. The mathematical literature concerning developables is quite extensive and has led to a complete characterization. Specifically, any developable surface must be the union of four fundamental types of developables: planar, tangent developable, generalized cones, and generalized cylinders. See do Carmo ([1], §4–2) and references cited therein for details.

Since any curved developable surface can be formed from a flat region while preserving the distance along any curve between any pair of points, the work required to deform thin sheets of materials as diverse as paper, leather, metal, and glass into developable shapes is much less than that needed to attain other shapes. Developable surfaces have thus been of longstanding relevance in many areas, including the design of ship hulls [2] and automobile parts [3,4], buildings [5,6], especially lightweight structures like facades [7] and pavilions [8], apparel [9] and footwear [10]. Due to the widespread utility of developable surfaces, methods for generating and processing them are now standard features of most computer-aided design platforms. In addition to devising algorithms for modelling the bending of paper and origami [11–13] and the draping and folding of cloth [14] for animation, computer scientists have discovered a host of novel applications for developable surfaces, including the segmentation and parametrization of meshes and the mapping of textures [15]. Very recently, mechanisms that can be placed on developable surfaces and, thus, allow for compact storage have been designed by Greenwood *et al.* [16] and Hyatt *et al.* [17].

Locally, a smooth developable surface can be represented as the envelope of a smoothly varying one parameter family of planes or as a ruled surface. See Section 2–4 of Struik [18] for a comprehensive discussion of these topics. Here we focus on the latter representation, whereby a smooth developable surface can be parametrized locally by a space curve, called its directrix, and a family of straight lines, called rulings or generators, emanating from that curve. Given a space curve, it is always possible to generate a ruled surface by attaching to it a smoothly varying family of rulings. However, not all ruled surfaces are developable. This leads naturally to the question of whether a developable surface can always be constructed from a given curve. The answer to this question is affirmative, as there are two well-known constructions: the tangent developable and the rectifying developable.

To describe these constructions, consider a smooth curve C with arclength parametrization $\text{d}:[a,b]\to R^{3}$. The tangent and rectifying developables generated by C are then parametrized by

1.1 $$\text{r}(\alpha ,\beta )=\text{d}(\alpha )+\beta {\text{d}}^{\prime }(\alpha ),\;(\alpha ,\beta )\in [a,b]\times R,$$

and

1.2 $$\text{r}(\alpha ,\beta )=\text{d}(\alpha )+\beta \frac{{\text{d}}^{\prime }(\alpha )\times {\text{d}}^{\prime \prime }(\alpha )+\tau (\alpha ){\text{d}}^{\prime }(\alpha )}{\sqrt{\kappa^{2}(\alpha )+\tau^{2}(\alpha )}},\;(\alpha ,\beta )\in [a,b]\times R,$$

where *κ* = |**d**″| and *τ* = (**d**′ × **d**″) · **d**″′/*κ*^2^ denote the curvature and torsion of C. Whereas the rulings of the tangent developable of C are aligned with the tangent of C, the rulings of the rectifying developable of C are aligned with the Darboux vector of C. Notice that tangent and rectifying developables may contain singular points and that a rectifying developable exists only if *κ* never vanishes.

Recall that the geodesic curvature *κ*_*g*_ of a curve C on a surface S measures how far C deviates from being a geodesic of S and that its magnitude |*κ*_*g*_| cannot exceed the curvature *κ* of C. The two classes of developables mentioned above manifest the extreme limiting cases of the inequality |*κ*_*g*_| ≤ *κ*. Relative to its tangent developable surface, the geodesic curvature of C satisfies |*κ*_*g*_| = *κ* on C, with the sign of *κ*_*g*_ being determined by the choice of the direction of the normal to the surface. Relative to its rectifying developable surface, the geodesic curvature of C satisfies *κ*_*g*_ = 0. In §3, we show that in addition to these two limiting cases, there is a continuous spectrum of developable surfaces associated with a curve C. Each such surface is generated by a function k:[a,b]→R satisfying |*k*| ≤ *κ*. Moreover, we show that the geodesic curvature *κ*_*g*_ of C relative to that surface obeys *κ*_*g*_ = *k* on C. To allow for situations in which the curvature *κ* of C vanishes on a finite number of intervals, we stipulate that *k* complies with a jump-like condition consistent with changes in orientation, about the tangent to C, of the Frenet frame at junctions between curved and straight segments of C. This is condition (C2) in theorem 3.1. An additional condition on *k*, (C3) in theorem 3.1, is needed to ensure a continuous transition of the rulings at any junction between segments of C within which |*k*| < *κ* and within which |*k*| = *κ*. However, we defer further mention of that condition until §3.

In §2, we review facts about curves in $R^{3}$ and curves on surfaces embedded in $R^{3}$, including the definitions of the Frenet and Darboux frames and the relationship between them. We also derive restrictions on the directions of the rulings associated with a curve on a developable surface. The purpose of deriving these restrictions is to motivative the assumptions underlying the main result established in §3, which states that given a curve C and a function *k* satisfying certain conditions which we delineate, it is possible to construct a developable surface S such that the geodesic curvature of C relative to S is *k*.

## 2. Curves on developables

Let C be a *C*^3^ curve in $R^{3}$ with arclength parametrization $\text{d}:[a,b]\to R^{3}$. At points where ${\text{d}}^{\prime \prime }\neq \text{0}$, the normal **p** and binormal **b** of the Frenet frame {**t**, **p**, **b**} for C are given by

2.1 $$\text{t}={\text{d}}^{\prime },\;\text{p}=\frac{{\text{d}}^{\prime \prime }}{|{\text{d}}^{\prime \prime }|}\;\text{and}\;\text{b}=\frac{{\text{d}}^{\prime }\times {\text{d}}^{\prime \prime }}{|{\text{d}}^{\prime \prime }|}.$$

The structure equations for the frame {**t**, **p**, **b**} are

2.2 $${\text{t}}^{\prime }=\kappa \text{p},\;{\text{p}}^{\prime }=-\kappa \text{t}+\tau \text{b},\;{\text{b}}^{\prime }=-\tau \text{p},$$

where

2.3 $$\kappa =|{\text{d}}^{\prime \prime }|\;\text{and}\;\tau =\frac{({\text{d}}^{\prime }\times {\text{d}}^{\prime \prime })\cdot {\text{d}}^{\prime \prime \prime }}{\kappa^{2}}$$

denote the curvature and torsion of C, respectively.

We assume that C is such that **d**″ = **0** on only a finite number of disjoint subintervals $\left[\alpha_{i}^{-},\alpha_{i}^{+}\right]$, *i* = 1, …, *n*, of (*a*, *b*) and that the limits

2.4 $${\text{p}}_{i}^{\pm }=\lim_{\varepsilon ↓0}\text{p}(\alpha_{i}^{\pm }\pm \varepsilon )\;\text{and}\;{\text{b}}_{i}^{\pm }=\lim_{\varepsilon ↓0}\text{b}(\alpha_{i}^{\pm }\pm \varepsilon )$$

exist. We allow, in particular, for the possibility that $\alpha_{i}^{-}=\alpha_{i}^{+}$, in which case the interval $\left[\alpha_{i}^{-},\alpha_{i}^{+}\right]$ collapses to a single point.

Let

2.5 $$K=\overset{n}{\underset{i=1}{⋃}}[\alpha_{i}^{-},\alpha_{i}^{+}]=\{\alpha \in [a,b]\;|\;\kappa (\alpha )=0\}$$

denote the union of the intervals upon which **d**″ = **0**. Then, the Frenet frame of C is not defined on K and the Frenet frames $\left\{{\text{t}}_{i},{\text{p}}_{i}^{-},{\text{b}}_{i}^{-}\right\}$ and $\left\{{\text{t}}_{i},{\text{p}}_{i}^{+},{\text{b}}_{i}^{+}\right\}$ at the two endpoints of each interval $\left[\alpha_{i}^{-},\alpha_{i}^{+}\right]$, *i* = 1, …, *n*, may differ. However, since $\left\{{\text{t}}_{i},{\text{p}}_{i}^{-},{\text{b}}_{i}^{-}\right\}$ and $\left\{{\text{t}}_{i},{\text{p}}_{i}^{+},{\text{b}}_{i}^{+}\right\}$ are both positively oriented for each *i* = 1, …, *n*, it is possible to rotate one of these frames around the axis determined by **t**_*i*_ to obtain the other. Thus, there is an angle *ψ*_*i*_ such that, for each *i* = 1, …, *n*,

2.6  pi+=−cos⁡ψi pi−+sin⁡ψi bi−, bi+=−sin⁡ψi pi−+cos⁡ψi bi−.}

In (2.6), positive values of *ψ*_*i*_ correspond to counterclockwise rotations about **t**_*i*_.

Suppose there is a *C*^2^ surface S that contains C and that {**t**, **m**, **n**} is a Darboux frame of C relative to S, with **n** normal to S and **m** = **n** × **t** tangent to S and orthogonal to C. The structure equations for the frame {**t**, **m**, **n**} are

2.7 $${\text{t}}^{\prime }=\kappa_{g}\text{m}+\kappa_{n}\text{n},\;{\text{m}}^{\prime }=-\kappa_{g}\text{t}+\tau_{g}\text{n},\;{\text{n}}^{\prime }=-\kappa_{n}\text{t}-\tau_{g}\text{m},$$

where *κ*_*g*_, *κ*_*n*_ and *τ*_*g*_, respectively, denote the geodesic curvature, normal curvature, and geodesic torsion of C relative to S. It follows from (2.3)_1_ and (2.7)_1_ that

2.8 $$\kappa^{2}=\kappa_{g}^{2}+\kappa_{n}^{2}.$$

Since the Frenet and Darboux frames of C are both positively oriented and share the same tangent direction **t**, it is possible to rotate the Frenet frame about the axis determined by **t** through a function *ϕ*:[*a*, *b*] → [0, 2*π*) to obtain the Darboux frame, so that

2.9  m=−cos⁡ϕ p+sin⁡ϕ b, n=−sin⁡ϕ p+cos⁡ϕ b.}

From (2.2)_1_, (2.7)_1_ and (2.9), *κ*_*g*_ and *κ*_*n*_ are related to *κ* and *ϕ* by

2.10 $$\kappa_{g}=\kappa \cos\phi \;\text{and}\;\kappa_{n}=-\kappa \sin\phi .$$

Moreover, from the consequence *τ*_*g*_ = **m**′ · **n** of (2.7)_2_ and (2.9), *τ*_*g*_ is related to *τ* and *ϕ* by

2.11 $$\tau_{g}=-(\kappa \cos\phi \;\text{t}+(\tau +\phi^{\prime })(\sin\phi \;\text{p}-\cos\phi \;\text{b}))\cdot (-\sin\phi \;\text{p}+\cos\phi \;\text{b})=\tau +\phi^{\prime }.$$

Since C is *C*^3^ and S is *C*^2^, the Darboux frame {**t**, **m**, **n**} of C relative to S is *C*^1^. Bearing in mind that the Frenet frames at the endpoints of an interval where **d**″ = **0** may differ and that the Darboux frame is continuous, it is not surprising that the angle *ϕ* that relates the Frenet and Darboux frames may also jump across these intervals. In fact, any such jump is related to *ψ*_*i*_. To verify this assertion, we first define the jump

2.12 $${\left[[\phi ]\right]}_{i}=\lim_{\varepsilon ↓0}(\phi (\alpha_{i}^{+}+\varepsilon )-\phi (\alpha_{i}^{-}-\varepsilon ))$$

of *ϕ* across the interval $\left[\alpha_{i}^{-},\alpha_{i}^{+}\right]$. Considering (2.9) evaluated at $\alpha =\alpha_{i}^{+}+\varepsilon$ and at $\alpha =\alpha_{i}^{-}-\varepsilon$ and using the continuity of **m**, the jump relations (2.6), and the sum formulae for sine and cosine, we next find that

2.13 $$\begin{array}{rl} & \sin(\lim_{\varepsilon ↓0}\phi (\alpha_{i}^{-}-\varepsilon ))=\sin(\lim_{\varepsilon ↓0}\phi (\alpha_{i}^{+}+\varepsilon )+\psi_{i}),\\ & \cos(\lim_{\varepsilon ↓0}\phi (\alpha_{i}^{-}-\varepsilon ))=\cos(\lim_{\varepsilon ↓0}\phi (\alpha_{i}^{+}+\varepsilon )+\psi_{i}). \end{array}\}$$

Since $\lim_{\varepsilon ↓0}\phi (\alpha_{i}^{-}-\varepsilon )$ and $\lim_{\varepsilon ↓0}(\phi (\alpha_{i}^{+}+\varepsilon )+\psi_{i})$ have the same sine and cosine, we see that they differ at most by a multiple of 2*π*, with the consequence that

2.14 $$\psi_{i}+{\left[[\phi ]\right]}_{i}\equiv 0\;\mathrm{mod}\;\;2\pi .$$

We now assume that S is developable. A basic property of such surfaces, a proof of which is provided in Section 2–4 of Struik [18], is that they are locally ruled. We therefore seek to represent a subset of the surface S in a neighbourhood of C as a ruled surface with directrix C. To carry out this construction, we use a result that Hartman & Nirenberg [19] first established for *C*^2^ surfaces and which was later shown by Pogorelov [20] to hold for surfaces with less regularity. The developable surface S can be partitioned into two subsets with specific geometric structure:

— A subset wherein the mean curvature does not vanish and, hence, S is curved. This part of S consists of connected components covered by straight line segments that do not intersect and are asymptotic curves.
— A subset wherein the mean curvature vanishes, and, hence, since the Gaussian curvature also vanishes, where S is flat. Any connected component of this subset of S is bounded by straight-line segments.

Let $S_{c}$ denote the curved subset of S and assume without loss of generality that $S_{c}\neq \emptyset$. From the stated result, there is a distinguished line segment, called a ruling, through every point on $S_{c}$. Since rulings cannot intersect, there exists a unit-vector valued function $\hat{\text{g}}$ defined on $S_{c}$ with the property that $\hat{\text{g}}(\text{z})$ is parallel to the ruling passing through $\text{z}\in S_{c}$. Invoking a result due to Hartman & Wintner [21], we infer that $\hat{\text{g}}$ is *C*^1^ on $S_{c}$. Moreover, recognizing that the boundary of $S_{c}$ consists of straight line segments, we see that $\hat{\text{g}}$ can be continuously extended to the closure ${\bar{S}}_{c}$ of $S_{c}$ relative to S.

Let A⊆[a,b] denote the subset of parameter values *α* ∈ [*a*, *b*] such that |*κ*_*g*_(*α*)| < *κ*(*α*). Since $\text{d}(\alpha )\in {\bar{S}}_{c}$ for all $\alpha \in \bar{A}$, we can define a function $\text{g}:A\to R^{3}$ such that

2.15 g(α)={−g^(d(α)),(d′(α)×g^(d(α)))⋅n(α)≥0,−g^(d(α)),(d′(α)×g^(d(α)))⋅n(α)<0,

for any α∈A, where $\hat{\text{g}}$ is the function mentioned in the previous paragraph. The motivation underlying the introduction of **g** will be explained in due course. To proceed, we first extend **g** from A to the entire interval [*a*, *b*] in such a way that:

— **g** is *C*^1^ on $[a,b]\setminus \bar{A}$,
— (**d**′ × **g**) · **n** ≥ 0, **g** · **n** = 0, and |**g**| = 1,
— **g** is continuous except possibly at points where it is parallel to **d**′.

We emphasize that **g** can be extended as described because $[a,b]\setminus \bar{A}$ consists of a collection of intervals that are open relative to [*a*, *b*]. Also, we observe that a feature of (2.15) is that **g** need not be continuous if it is parallel to **d**′.

Consider, next, the parametrization $\text{r}:[a,b]\times (-\bar{\beta },\bar{\beta })\to R^{3}$ defined such that

2.16 $$\text{r}(\alpha ,\beta )=\text{d}(\alpha )+\beta \text{g}(\alpha ),\;(\alpha ,\beta )\in [a,b]\times (-\bar{\beta },\bar{\beta }).$$

Since **g** is parallel to the rulings on S, the range of **r** is a subset of S for any sufficiently small choice of $\bar{\beta }$. Moreover, since **g** is tangent to S and is of unit length, there exists a function *θ*:[*a*, *b*] → [0, *π*] such that

2.17 g=cos⁡θ t+sin⁡θ m.

By the construction of **g**, we see that *θ* is continuous except possibly if sin*θ* = 0, in which case it may jump by *π*. Differentiating **g** as defined by (2.17) and using the structure equations (2.7) for the Darboux frame, we find that

2.18 $${\text{g}}^{\prime }=-(\theta^{\prime }+\kappa_{g})(\sin\theta \;\text{t}-\cos\theta \;\text{m})+(\kappa_{n}\cos\theta +\tau_{g}\sin\theta )\text{n}.$$

Recalling that a surface with parametrization **r** has a singular point at (*α*, *β*) if ${\text{r}}_{\alpha }\times {\text{r}}_{\beta }$ evaluated at (*α*, *β*) vanishes, we see with reference to (2.16) and (2.18) that (*α*, *β*) is a singular point if

2.19 $$\sin\theta (\alpha )-\beta (\theta^{\prime }(\alpha )+k(\alpha ))=0.$$

At any point where (2.19) does not hold, there exists a neighbourhood within which **r** is locally invertible and, hence, provides a chart of S. We thus conclude from (2.16) to (2.18) that, at any non-singular point of the surface S with parametrization **r** defined in (2.16), the Gaussian curvature *K* can be expressed as

2.20 $$K=-\frac{{\left(\kappa_{n}\cos\theta +\tau_{g}\sin\theta \right)}^{2}}{{\left(\sin\theta -\beta (\theta^{\prime }+\kappa_{g})\right)}^{2}+\beta^{2}{\left(\kappa_{n}\cos\theta +\tau_{g}\sin\theta \right)}^{2}}.$$

However, since *K* = 0 on S, we see from (2.20) that *κ*_*n*_, *τ*_*g*_, and *θ* must obey the condition

2.21 $$\kappa_{n}\cos\theta +\tau_{g}\sin\theta =0.$$

If |*κ*_*g*_| < *κ*, we may use (2.10)_2_ and (2.11) in (2.21) to find that

2.22 $$\kappa \cos\phi \cos\theta +(\tau +\phi^{\prime })\sin\theta =0.$$

Bearing in mind that sin*ϕ* = 0 away from A, we thus see from (2.22) that the angle *θ* introduced in (2.17) is given on A by

2.23 $$\cot\theta =\frac{\tau +\phi^{\prime }}{\kappa \sin\phi }.$$

However, because |cot⁡θ| is continuous on [*a*, *b*], we also see that the limit

2.24 $$\lim_{\begin{array}{c} \alpha \to \alpha_{\circ }\\ \alpha \in A \end{array}}|\frac{\tau (\alpha )+\phi^{\prime }(\alpha )}{\kappa (\alpha )\sin\phi (\alpha )}|,\;\alpha_{\circ }\in \bar{A},$$

must always exist but may be infinite.

Since sin*ϕ* = 0 and *ϕ* is *C*^1^ on $\left[a,b]\setminus (\bar{A}\cup K\right)$, we infer that *ϕ*′ = 0 on $\left[a,b]\setminus (\bar{A}\cup K\right)$ and, thus, from (2.10)_2_, (2.11), and (2.21), that *τ* and *θ* must satisfy

2.25 $$\tau \sin\theta =0,\;\text{on}\;[a,b]\setminus (\bar{A}\cup K).$$

The condition (2.25) is consistent with the observation that if |*κ*_*g*_| = *κ*, then the corresponding surface S is the union of two types of regions:

— planar regions generated by the curve C if *τ* = 0, or
— tangent developable regions where sin*θ* = 0.

It is possible for part of the surface S to be planar and a tangent developable simultaneously. The conjunction ‘or’ used above is therefore not to be taken in the exclusive sense.

The parameter set T defined by

2.26 $$T=\{\alpha \in [a,b]\setminus (\bar{A}\cup K)\;|\;\tau (\alpha )\neq 0\}$$

consists of all choices of $\alpha \in [a,b]\setminus (\bar{A}\cup K)$ at which S is a tangent developable. To ensure a continuous transition of *θ* from T to A, *θ* must be such that sin*θ* = 0 on $\partial \bar{A}\cap \partial T$. Using (2.24), we may formulate this condition as

2.27 $$\alpha_{\circ }\in \partial \bar{A}\cap \partial T\;\text{implies that}\;\lim_{\begin{array}{c} \alpha \to \alpha_{\circ }\\ \alpha \in A \end{array}}|\frac{\tau (\alpha )+\phi^{\prime }(\alpha )}{\kappa (\alpha )\sin\phi (\alpha )}|=\infty .$$

The calculations in this section demonstrate that a space curve cannot lie on a developable surface unless its curvature *κ* and geodesic curvature *κ*_*g*_ relative to that surface satisfy certain conditions. First, it follows from (2.8) that *κ*_*g*_ must satisfy |*κ*_*g*_| ≤ *κ*. Second, if (2.10)_1_ is interpreted as a relation that determines *ϕ* as a function of *κ* and *κ*_*g*_, then *κ* and *κ*_*g*_ must satisfy the jump-like condition (2.14). Finally, the limit in (2.24) must exist and is infinite if $\alpha_{\circ }\in \partial \bar{A}\cap \partial T$. These three conditions on *κ* and *κ*_*g*_ must hold if the surface containing the curve is developable. It is these conditions that motivate the assumptions appearing in theorem 3.1 in the next section.

An alternative to starting with a developable surface S and constructing a ruled parametrization of the form (2.16) for S in a neighbourhood of C involves starting with a ruled surface S of vanishing Gaussian curvature *K*. This option has the advantage of rendering the discussion in the paragraphs between (2.14) and (2.16) unnecessary, as it makes it possible to begin directly with the parametrization (2.16). It also resembles more closely the construction described in §3. However, starting with a ruled surface has the potential disadvantage of conveying the false impression that all developable surfaces are globally, as opposed to locally, ruled.

## 3. Construction of a family of developable surfaces

The main idea underpinning the theorem that we state and prove below is that, given a curve C and a function *k* satisfying certain conditions, it is possible to generate a ruled surface S with vanishing Gaussian curvature *K* such that C lies on S and has geodesic curvature *κ*_*g*_ equal to *k*. We emphasize that a ruled surface S generated in this manner need not be smooth.

**Theorem 3.1.**

*Let* $\text{d}:[a,b]\to R^{3}$ *be an arclength parametrization of a* *C*^4^ *space curve* C *with curvature* *κ* *that vanishes on at most a finite number of intervals* $\left[\alpha_{i}^{-},\alpha_{i}^{+}\right]$, *i* = 1, …, *n*. *Consider a* *C*^2^ *function* k:[a,b]∖K→R, *where* K *is defined in* (2.5), *and let* A *be the subset of* [*a*, *b*] *defined by*

3.1 A={α∈[a,b]∖K | |k(α)|<κ(α)}.

*Assume that*:

— |*k*| ≤ *κ*;
— *the function* ϕ:[a,b]∖K→[0,π] *defined by*
  3.2 $$\phi =\arccos\frac{k}{\kappa }$$
  *satisfies*
  3.3 $$\psi_{i}+{\left[[\phi ]\right]}_{i}\equiv 0\;\mathrm{mod}\;\;2\pi ,\;i=1,\ldots ,n,$$
  *where* *ψ*_*i*_ *is defined so that* (2.6) *holds*;
— *the not necessarily finite limit*
  3.4 $$\lim_{\begin{array}{c} \alpha \to \alpha_{\circ }\\ \alpha \in A \end{array}}|\frac{\tau (\alpha )+\phi^{\prime }(\alpha )}{\kappa (\alpha )\sin\phi (\alpha )}|,\;\alpha_{\circ }\in \partial A,$$
  *exists and is infinite if* $\alpha_{\circ }\in \partial \bar{A}\cap \partial T$, *with* T *as in* (2.26).

*Then there exists a ruled surface* S, *containing* C, *with continuously varying rulings such that the geodesic curvature* *κ*_*g*_ *of* C *relative to* S *is given by* *k* *and the Gaussian curvature of* S *vanishes at all non-singular points of* S.

Before proving theorem 3.1, we provide interpretations of conditions (C2) and (C3). Condition (C2) ensures that the Darboux frame constructed from the space curve C has a continuous extension across the intervals where *κ* = 0. If *κ* never vanishes, then (C2) is unnecessary to obtain the result. Condition (C3) is related to the direction of the rulings. Unique rulings are generated from C if |*k*| < *κ*. As |*k*| → *κ*, condition (C3) guarantees that the rulings approach a particular direction. If |*k*| is never equal to *κ*, then (C3) is unnecessary since in this case ∂A is empty.

**Proof.**

We begin by using *ϕ* to define **m** and **n** on [a,b]∖K by

3.5  m=−cos⁡ϕ p+sin⁡ϕ b, n=−sin⁡ϕ p+cos⁡ϕ b.}

We then see from (2.2) that

3.6 $${\text{m}}^{\prime }=-k\;\text{t}+(\tau +\phi^{\prime })\;\text{n}.$$

Since (3.3) holds, we recognize that on each interval $\left[\alpha_{i}^{-},\alpha_{i}^{+}\right]$, *i* = 1, …, *n*, upon which *κ* = 0 the jumps [[**m**]]_*i*_ and [[**n**]]_*i*_ of **m** and **n** vanish:

3.7 $${\left[[\text{m}]\right]}_{i}=0\;\text{and}\;{\left[[\text{n}]\right]}_{i}=0,\;i=1,\ldots ,n.$$

We may therefore extend **m** and **n** so that they are constant on the intervals $\left[\alpha_{i}^{-},\alpha_{i}^{+}\right]$, *i* = 1, …, *n*, with the consequence that they become continuous on the entire interval [*a*, *b*].

Next, we define *θ* implicitly on A through the relation

3.8 $$\cot\theta =\frac{\tau +\phi^{\prime }}{\kappa \sin\phi }.$$

It then follows from the assumed regularity of C and *k* and the implicit function theorem that *θ* is *C*^1^ on A. By (C3), we may use (3.8) to define *θ* for α∈∂A, where we choose *θ* = 0 if the limit in (C3) is infinite. Using this definition, *θ* may jump by *π* if *θ* = 0, but otherwise is continuous on $\bar{A}$. We now seek to define *θ* on the remainder $[a,b]\setminus \bar{A}$ of [*a*, *b*] such that

3.9 $$\tau \sin\theta =0,\;\text{on}\;[a,b]\setminus (\bar{A}\cap K).$$

Since $[a,b]\setminus \bar{A}$ is open relative to [*a*, *b*], it can be expressed as the union of a countable collection of intervals that are open relative to [*a*, *b*]. Let (*α*_−_, *α*_+_) be one such interval. Notice that *θ* is already defined at the endpoints *α*_±_ of that interval. Situations in which the interval under consideration is of the semi-open form [*a*, *α*_+_) or (*α*_−_, *b*] will not be discussed in detail here. However, the strategy described below is easily adapted to treat those situations. Recalling the definition of T in (2.26), the closure of the convex hull of $T\cap [\alpha_{-},\alpha_{+}]$ is an interval, which we denote by $\left[\alpha_{-}^{\tau },\alpha_{+}^{\tau }\right]$. We set *θ* = 0 on $\left(\alpha_{-}^{\tau },\alpha_{+}^{\tau }\right)$. If $\alpha_{-}=\alpha_{-}^{\tau }$ and $\alpha_{+}=\alpha_{+}^{\tau }$, then by (C3) *θ* is continuous on [*α*_−_, *α*_+_] and *C*^1^ on (*α*_−_, *α*_+_). However, if $\alpha_{-}<\alpha_{-}^{\tau }$, then it is always possible to define *θ* on the interval $\left(\alpha_{+},\alpha_{+}^{\tau }\right)$ so as to ensure that it is *C*^1^ on (*α*_−_, *α*_+_). A similar argument holds if $\alpha_{+}^{\tau }<\alpha_{+}$. Moreover, this construction can be performed on each interval that comprises $[a,b]\setminus \bar{A}$. To summarize, *θ* is now defined on the entire interval [*a*, *b*], is *C*^1^ away from ∂A, is continuous except possibly if *θ* = 0, where only a jump of *π* may occur, and (3.9) holds.

With this definition of *θ*, we next construct **g** through the relation

3.10 g=cos⁡θ t+sin⁡θ m.

Using (2.2), (3.6) and (3.10), we find that

3.11 $${\text{g}}^{\prime }=-(\theta^{\prime }+k)(\sin\theta \;\text{t}-\cos\theta \;\text{m})+((\tau +\phi^{\prime })\sin\theta -\kappa \sin\phi \cos\theta )\text{n}$$

on A. However, since *θ* satisfies (3.8) on A, we see that (3.11) reduces to

3.12 $${\text{g}}^{\prime }=-(\theta^{\prime }+k)(\sin\theta \;\text{t}-\cos\theta \;\text{m}).$$

Moreover, on $\left[a,b]\setminus (\bar{A}\cup K\right)$, where |*k*| = *κ*, we see that sin*ϕ* = 0 and *ϕ*′ = 0, whereby (3.11) reduces to

3.13 $${\text{g}}^{\prime }=-(\theta^{\prime }+k)(\sin\theta \;\text{t}-\cos\theta \;\text{m})+\tau \sin\theta \;\text{n}.$$

However, we see from (3.9) that (3.13) reduces further to coincide with (3.12). Finally, since **t** and **m** are constant on any open interval where *κ* = 0, we see that on such intervals

3.14 $${\text{g}}^{\prime }=-\theta^{\prime }(\sin\theta \;\text{t}-\cos\theta \;\text{m}).$$

We hence find that whenever **g**′ exists it satisfies

3.15 $${\text{g}}^{\prime }\cdot \text{n}=0.$$

Next, we define $\text{r}:[a,b]\times R\to R^{3}$ by

3.16 r(α,β)=d(α)+βg(α),(α,β)∈[a,b]×R.

Due to the assumed regularity of **d** and *k* and the regularity of *θ* as constructed above, **r** as defined in (3.16) is *C*^1^ on ([a,b]∖∂A)×R. Moreover, the rulings of the surface S parametrized by **r** vary continuously with *θ* in the sense that the projector **g** ⊗ **g** in the direction of **g** is continuous because *θ* can only jump by *π* if sin*θ* = 0. This property ensures that the surface parametrized by **r** exhibits no jumps.

Notice from (3.12) and (3.16) that

3.17 $${\text{r}}_{\alpha }\times {\text{r}}_{\beta }=(\text{t}+\beta {\text{g}}^{\prime })\times \text{g}=(\sin\theta -\beta (\theta^{\prime }+k))\text{n}$$

and, hence, that the surface S parametrized by **r** is singular if

3.18 $$\sin\theta -\beta (\theta^{\prime }+k)=0$$

or if the necessary derivatives do not exist. Moreover, we see from (3.17) that, at non-singular points, the normal to S is given by

3.19 $$\frac{{\text{r}}_{\alpha }\times {\text{r}}_{\beta }}{|{\text{r}}_{\alpha }\times {\text{r}}_{\beta }|}=\text{n}$$

and, hence, is independent of *β*. The Gaussian curvature *K* of S can therefore be calculated as in (2.20). With reference to (3.15), we find that *K* vanishes at all non-singular points of S.

Finally, we use (2.7)_2_ and (3.6) to find that

3.20 $$\kappa_{g}=-{\text{m}}^{\prime }\cdot \text{t}=k,$$

and, hence, that the geodesic curvature *κ*_*g*_ of the curve C relative to the surface S parametrized by **r** is given by the function *k*.  □

The main steps in the construction of a developable surface S laid out in theorem 3.1 can be synopsized as follows:

— Use (3.2) to define the function *ϕ*:[*a*, *b*] → [0, *π*].
— Use (3.5) to define the Darboux frame {**t**, **m**, **n**} from the Frenet frame {**t**, **p**, **b**} associated with **d**. Notice, here, that (C2) ensures that {**t**, **m**, **n**} is continuous.
— If |*k*| < *κ*, use (3.8) to define the function *θ*:[*a*, *b*] → [0, *π*]. Otherwise, if there are parameter values *α* for which |*k*(*α*)| = *κ*(*α*), define *θ* on the proper subset A of [*a*, *b*] upon which |*κ*_*g*_| < *κ* and extend it to [*a*, *b*] so that *τ*(*α*)sin*θ*(*α*) = 0 at each α∈[a,b]∖A for which |*k*(*α*)| = *κ*(*α*). Notice that, for the second of these alternatives, (C3) ensures that the method of extension results in sin*θ* being continuous on [*a*, *b*].
— Use (3.10) to define the orientation **g** of the rulings.
— Use (3.16) to define the parametrization **r** of the developable surface S.

If |*k*| is never equal to *κ* and *κ* never vanishes, then conditions (C2) and (C3) are vacuously satisfied and the construction of a corresponding developable surface S greatly simplifies. In particular, (3.2) and (3.8) can be used to express **g** directly in terms of *k*, the curvature *κ* and torsion *τ* of the curve C, and its Frenet frame:

3.21 $$\text{g}=\frac{\left(\tau +(k\kappa^{\prime }-k^{\prime }\kappa )/\kappa \sqrt{\kappa^{2}-k^{2}}\right)\text{t}+\left(k\sqrt{\kappa^{2}-k^{2}}/\kappa \right)\text{p}+((\kappa^{2}-k^{2})/\kappa )\text{b}}{\sqrt{(\tau +(k\kappa^{\prime }-k^{\prime }\kappa )/\kappa \sqrt{\kappa^{2}-k^{2}}{)}^{2}+\kappa^{2}-k^{2}}}.$$

Notice that theorem 3.1 is predicated on the assumption that the curvature of the curve C only vanishes on subintervals of [*a*, *b*] and, hence, does not apply if C is a straight-line segment. However, given such a curve, the only possible choice of *k* consistent with (C1) is the zero function and any plane containing C is a developable surface relative to which the geodesic curvature of C vanishes.

## 4. Examples

For illustrative purposes, we consider a helical curve H of radius *ρ* and pitch 2*πμ*, with arclength parametrization

**Figure 1.**
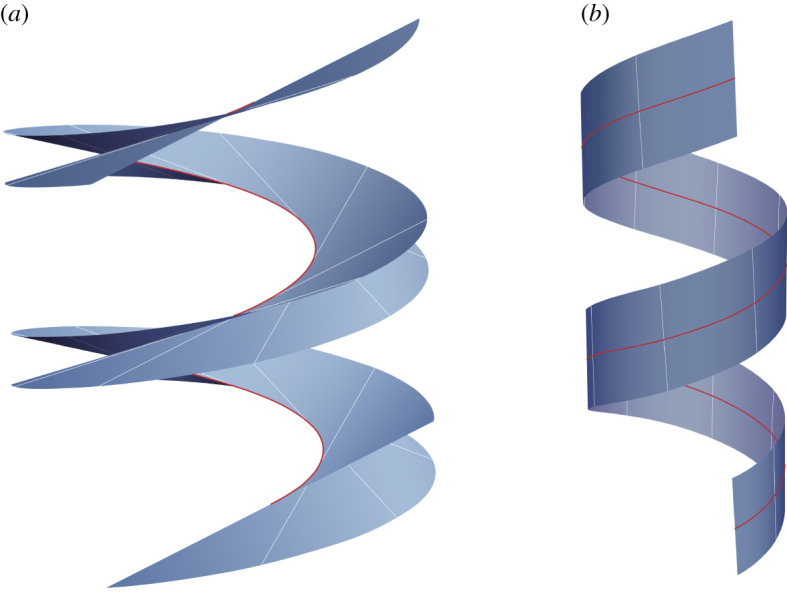
A depiction of the (*a*) tangent developable and (*b*) rectifying developable associated with the helix parametrized by (4.1) with the choice *ρ*/*μ* = 3. For the tangent developable *β* ranges over [ − 2*ρ*, 2*ρ*], while for the rectifying developable it ranges over [ − *ρ*/2, *ρ*/2].

4.1 $$\text{d}(\alpha )=\left(\rho \cos\frac{\alpha }{\sqrt{\rho^{2}+\mu^{2}}},\rho \sin\frac{\alpha }{\sqrt{\rho^{2}+\mu^{2}}},\frac{\mu \alpha }{\sqrt{\rho^{2}+\mu^{2}}}\right),\;\alpha \in [0,l]$$

relative to the canonical Cartesian basis. For the length *l* of H, we choose $l=4\pi \sqrt{\rho^{2}+\mu^{2}}$, so that H has only two turns. The tangent developable and rectifying developable surfaces associated with H are shown in figure 1. To generate the tangent developable, we set *k* = *κ* = *ρ*/(*ρ*^2^ + *μ*^2^). According to (3.1), A is empty for this choice of *k*. Since, from the construction outlined above, *θ* must be chosen such that *τ*sin*θ* = 0 away from A, and since *τ* never vanishes for a helix, it is permissible to set *θ* = 0. Moreover, since *ϕ* = 0 if *k* = *κ* as a consequence of (3.2), we find from (3.5) and (3.10) that **g** = **t** and, hence, that the parametrization of the surface generated from the choice of *k* under consideration is (1.1), the parametrization of the tangent developable. From figure 1*a*, it is evident that the tangent developable consists of two sheets that meet and are tangent along the generating curve. Struik [18] shows that this is a property common to all tangent developables. The tangency of the two sheets ensures that the geodesic curvature of the generating curve is the same relative to each sheet and, thus, is well defined. If, instead, *k* = 0, then A is the entire interval [0, *l*], so we may use (3.21) with *k* = 0 to find that

4.2 $$\text{g}=\cos\theta \;\text{t}+\sin\theta \;\text{b}=\frac{\tau \text{t}+\kappa \text{b}}{\sqrt{\kappa^{2}+\tau^{2}}},$$

whereby the surface generated from the choice *k* = 0 coincides with the rectifying developable parametrized by (1.2).

Any function k:[0,l]→R that satisfies the conditions (C1)–(C3) of our theorem 3.1 can be used to generate a developable surface. Two non-trivial choices are

4.3 $$\begin{array}{rl} & k(\alpha )=\frac{\rho }{\rho^{2}+\mu^{2}}\sin\left(\frac{6\alpha -3l}{2l}\right),\\ & k(\alpha )=\frac{20\rho \alpha (\alpha -l/2)(\alpha -l)}{l^{3}(\rho^{2}+\mu^{2})}, \end{array}\}\;\alpha \in [0,l].$$

Since *κ* = *ρ*/(*ρ*^2^ + *μ*^2^), it is readily seen that the *k* defined in (4.3)_1_ satisfies |*k*| < *κ*. It can be shown that the minimum and maximum values of the *k* defined in (4.3)_2_ are

**Figure 2.**
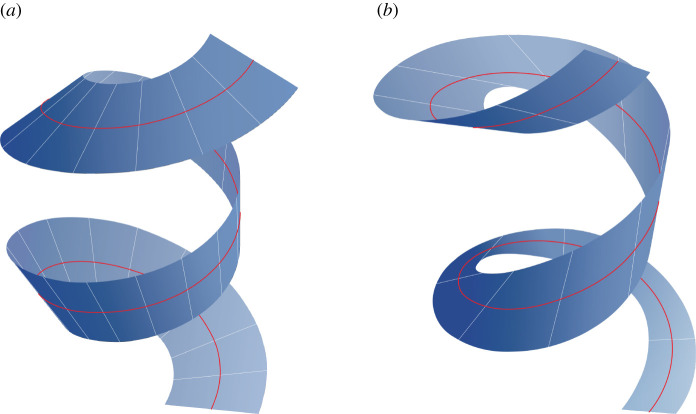
Depiction of the developable surfaces associated with the helix parametrized by (4.1) and the functions *k* defined in (*a*) (4.3)_1_ and (*b*) (4.3)_2_ both with the choice *ρ*/*μ* = 3. The surfaces were generated with *β* ranging over [ − *ρ*/2, *ρ*/2].

4.4 $$\pm \frac{5\rho }{3\sqrt{3}(\rho^{2}+\mu^{2})}≃\pm \frac{0.96\rho }{\rho^{2}+\mu^{2}}$$

and, hence, that this choice of *k* also satisfies |*k*| < *κ*. Thus, (C1) holds for both choices in (4.3). Moreover, since *κ* never vanishes, (C2) holds. Finally, because |*k*| is never equal to *κ*, (C3) is vacuously satisfied. The developable surfaces associated with these choices of *k* are shown in figure 2.

To illustrate the importance of (C2), we consider a curve L parametrized by

4.5 $$\delta (t)=\left\{ \begin{array}{ll} (t,\frac{t^{5}}{2},0), & t\in [-1,0],\\ (t,0,\frac{t^{5}}{2}), & t\in (0,1]. \end{array}\right.$$

Notice that **δ** is *C*^4^ but does not provide an arclength parametrization of L. We denote the length of L by *l* and its arclength parametrization by $\text{d}:[-l/2,l/2]\to R^{3}$. Notice that L is planar over the interval [ − *l*/2, 0) and, thus, that *τ* = 0 on [ − *l*/2, 0). Similarly, L is planar over (0, *l*/2] and *τ* = 0 on (0, *l*/2] as well. With reference to (2.6), we see that the Frenet frame of L jumps at *α* = 0 by a rotation of *ψ* = *π*/2.

According to (C2) in theorem 3.1, we may generate a developable surface from **d** by selecting *k* such that *ϕ*, as defined in (3.2), has a jump discontinuity at *α* = 0. The choice

4.6 $$k(\alpha )=\left\{ \begin{array}{ll} -\frac{\kappa (\alpha )}{2}, & \alpha \in [-\frac{l}{2},0),\\ \frac{\sqrt{3}\kappa (\alpha )}{2}, & \alpha \in (0,\frac{l}{2}]. \end{array}\right.$$

results in a *ϕ* with the desired property. It can be shown that this choice of *k* also satisfies (C1) and (C3). Notice, in addition, that *ϕ*′(*α*) = 0 if α≠0. We thus find that the ratio

**Figure 3.**
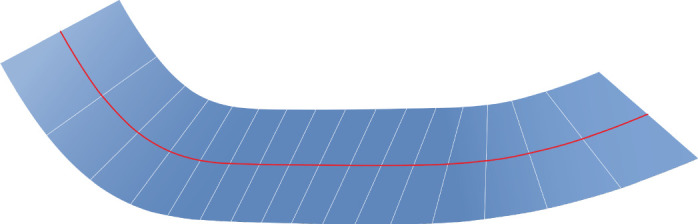
Depiction of the developable surface associated with the curve parametrized by (4.5) and the function *k* defined in (4.6). The plot was generated with *β* ranging over [ − 1/8, 1/8].

4.7 $$\frac{\tau (\alpha )+\phi^{\prime }(\alpha )}{\kappa (\alpha )\sin\phi (\alpha )}$$

vanishes if α≠0. Adhering to the construction of the developable surface in theorem 3.1, we should use (3.8) and set *θ*(*α*) = *π*/2 for all *α* ∈ [ − *l*/2, *l*/2]. The parametrization of the surface can then be defined on invoking (2.9), (2.16), and (2.17). See figure 3 for a plot of this surface.

In each example presented so far, condition (C3) holds vacuously. To demonstrate that condition (C3) is necessary for the construction of a developable surface from a curve, we consider a semicircle C scaled to be of unit radius. An arclength parametrization of C is given by

4.8 $$\text{d}(\alpha )=(\cos\alpha ,\sin\alpha ,0),\;\alpha \in \left[-\frac{\pi }{2},\frac{\pi }{2}\right].$$

Introducing a function *k* through the definition

4.9 $$k(\alpha )=\left\{ \begin{array}{ll} 1+\alpha^{5}(1-\sin\frac{1}{\alpha }), & \alpha \in [-\frac{\pi }{2},0),\\ 1, & \alpha =0,\\ 1-\alpha^{5}(1+\sin\frac{1}{\alpha }), & \alpha \in (0,\frac{\pi }{2}]. \end{array}\right.$$

we confirm that it is *C*^2^ and satisfies both (C1) and (C2). However, we see that (C3) does not hold. To verify this finding, begin by observing that *k* achieves a local maximum *k*(*α*_*n*_) = 1 at

4.10 $$\alpha_{n}=\frac{1}{2\pi n+3\pi /2},\;n\in N.$$

Since *k* is *C*^2^ and is not constant on (*α*_*n*+1_, *α*_*n*_), we may use the mean-value theorem to show that there exists *β*_*n*_ in this interval such that *k*′(*β*_*n*_) = 0. We thus infer that *ϕ*′(*β*_*n*_) = 0 and, hence, that

4.11 $$\frac{\tau (\beta_{n})+\phi^{\prime }(\beta_{n})}{\kappa (\beta_{n})\sin\phi (\beta_{n})}=0,\;n\in N.$$

If, alternatively, we set *γ*_*n*_ = 1/(2*πn* + *π*/4), for n∈N, we find that

4.12 $$\frac{\tau (\gamma_{n})+\phi^{\prime }(\gamma_{n})}{\kappa (\gamma_{n})\sin\phi (\gamma_{n})}=-\frac{2\sqrt{2}-10(2+\sqrt{2})\gamma_{n}}{4(2+\sqrt{2})\gamma_{n}^{2}-(3+2\sqrt{2})\gamma_{n}^{7}}$$

and, hence, deduce that

4.13 $$\lim_{n\to \infty }\frac{\tau (\gamma_{n})+\phi^{\prime }(\gamma_{n})}{\kappa (\gamma_{n})\sin\phi (\gamma_{n})}=-\infty .$$

Since $\beta_{n}\in A$ and $\gamma_{n}\in A$, the limit in (C3) at *α* = 0 does not exist. Based on the above calculations, we see that the function *θ* constructed from the choice of *k* defined in (4.9) oscillates rapidly between 0 and *π*/2 as *α* approaches 0 and, hence, is not continuous at this point. Thus, this choice of *k* cannot be used to generate a developable surface from the semicircular curve C defined via (4.8).

## 5. Discussion

The ability to construct a developable surface S from a space curve C with curvature *κ* and a given function *k* satisfying |*k*| ≤ *κ* has become important because of recent developments in the study of unstretchable two-dimensional sheets, which can only sustain isometric deformations. If the reference configuration D of such a sheet is planar, then after deformation its configuration must be a developable surface S. Granted that the bending energy density depends quadratically on the second fundamental form of S and that the sheet is isotropic, the total dimensionless bending energy of the sheet in the configuration S must have the form

5.1 $$\int_{S}H^{2}\;\text{d}a,$$

where *H* is the mean curvature of S. In a quest to find the equilibrium configurations of a half-twist Möbius band made from a rectangular strip, Wunderlich [22,23] showed that S must lie on a rectifying developable of its midline, which serves as the directrix C, and that (5.1) can be written as a single integral over C.

To expand this analysis to the problem of isometrically deforming a non-rectangular planar region D, it is necessary to take a different approach since D need not have a well-defined midline. Instead, the boundary of D is a natural, intrinsic choice for a referential base curve. After isometrically deforming D to a curved surface S, the boundary C=∂S of S need not be a geodesic of S. It must, however, have geodesic curvature equal to the signed curvature *k* of ∂D since the underlying deformation is isometric. In a future work, we will demonstrate that the bending energy (5.1) can be written as a single integral over C. For this new dimensionally reduced bending energy to be a viable substitute for (5.1), it must be possible to construct a developable surface from the curve C=∂S with geodesic curvature *k*. Theorem 3.1, which is stated and proved in this paper demonstrates that this is possible.

## Data accessibility

All of the data are available within the paper.
