# Supplementary material for: Bridging the gap between rectifying developables and tangent developables: a family of developable surfaces associated with a space curve
Source: Proc Math Phys Eng Sci. 2021 Feb 10;477(2246):20200617. doi: 10.1098/rspa.2020.0617 (PMC8317982; doi:10.1098/rspa.2020.0617)
Supplement: Supplementary file 1 [file rspa20200617_review_history.pdf]

THE ROYAL SOCIETY
PUBLISHING

PROCEEDINGS A

Bridging the gap between rectifying developables and tangent developables: a family of developable surfaces associated with a space curve

Brian Seguin, Yi-chao Chen and Eliot Fried

Article citation details
Proc. R. Soc. A **477**: 20200617.
http://dx.doi.org/10.1098/rspa.2020.0617

Review timeline

Original submission:	30 July 2020	
Revised submission:	12 January 2021	
Final acceptance:	13 January 2021	

Note: Reports are unedited and appear as submitted by the referee. The review history appears in chronological order.

Review History

RSPA-2020-0617.R0 (Original submission)

Review form: Referee 1

Is the manuscript an original and important contribution to its field?
Good

Is the paper of sufficient general interest?
Good

Is the overall quality of the paper suitable?
Good

Can the paper be shortened without overall detriment to the main message?
Yes

Do you think some of the material would be more appropriate as an electronic appendix?
Yes

Do you have any ethical concerns with this paper?
No

Reports © 2021 The Reviewers; Decision Letters © 2021 The Reviewers and Editors; Responses © 2021 The Reviewers, Editors and Authors. Published by the Royal Society under the terms of the Creative Commons Attribution License http://creativecommons.org/licenses/by/4.0/, which permits unrestricted use, provided the original author and source are credited

Recommendation?
Accept with minor revision (please list in comments)

Comments to the Author(s)
From a space curve, there are two familiar constructions of a developable surface. The tangent developable whose rulings are tangent to the curve at each point and relative to the surface the absolute value of geodesic curvature of the curve is equal to the curvature of the curve. The other construction is the rectifying developable. The geodesic curvature of the curve relative to this surface vanishes. The authors show that there is a family of developable surfaces that can be generated from a curve, one surface for each function k that is defined on the curve and satisfies $|k| < \kappa$, and that the geodesic curvature of the curve relative to each such constructed surface satifies $k_g \leq k$. The theoretical background is interesting. If the authors explain the equations (2.12) and (2.13) in detail or explain them by pictures, I think it will be more acceptable.

Review form: Referee 2

Is the manuscript an original and important contribution to its field?
Good

Is the paper of sufficient general interest?
Good

Is the overall quality of the paper suitable?
Good

Can the paper be shortened without overall detriment to the main message?
Yes

Do you think some of the material would be more appropriate as an electronic appendix?
No

Do you have any ethical concerns with this paper?
No

Recommendation?
Accept with minor revision (please list in comments)

Comments to the Author(s)
I recommend the paper "Bridging the gap between rectifying developables and tangent developables: a family of developable surfaces associated with a space curve" by S. Brian, Y.-C. Chen and E. Fried for publication after considering my comments below.

---Please consider to include two other very recent papers on developable surfaces:

O. Stein, E. Grinspun and K. Crane:
Developability of Triangle Meshes.
ACM Trans. Graph, 37(4), 2018.

M. Rabinovich, T. Hoffmann and O. Sorkine-Hornung:
The Shape Space of Discrete Orthogonal Geodesic Nets,
ACM Trans. Graph, 37(6), 2018.

---The authors use the notion "pointwise vanishing ..." throughout the paper. I would appreciate if you could rephrase it. For example, I would say that minimal surfaces have vanishing mean curvature and I would not say that minimal surfaces have *pointwise* vanishing mean curvature.

---In "classical" differential geometry, a curve, parametrized by d, is *regular* iff d' is not vanishing. However, the authors introduce the notion of "piecewise regular" (line 40) by d'' = 0 only on a finite number of disjoint subintervals. That is a bit confusing notion. Can you change that?

---line 49: I think you are talking about a ruled surface with vanishing mean curvature. However, such a surface does not necessarily have vanishing Gaussian curvature as the helicoid is a counterexample. And therefore, S is not necessarily flat. Please reconsider that statement.

---Why does Struik (1961) not get a number in the Reference section?

Review form: Referee 3

Is the manuscript an original and important contribution to its field?
Excellent

Is the paper of sufficient general interest?
Good

Is the overall quality of the paper suitable?
Excellent

Can the paper be shortened without overall detriment to the main message?
Yes

Do you think some of the material would be more appropriate as an electronic appendix?
No

Do you have any ethical concerns with this paper?
Yes

Recommendation?
Accept with minor revision (please list in comments)

Comments to the Author(s)
This is a positive review of the article entitled
Bridging the gap between rectifying developables and tangent developables: a family of developable surfaces
associated with a space curve written by Seguin, Chen and Fried.

Publication in Proc. Royal Soc. A is recommended without reservation.

This submission demonstrates the existence of a continuum of developables built from a single space curve, including the tangent developable and rectifying developable as outliers within the collection. It appears to be an infinite dimensional family, as it is parametrized by a family of functions.

There is a thorough explanation of historical context and applications, which indicates why the authors are interested in fixing the initial curve for constructing the surfaces, and why the curve

is allowed to have intervals of zero curvature, and why the surfaces are not assumed to be C-infinity smooth. Ambiguity for the Frenet frame results at points where the initial curve has curvature zero. Mathematicians generally resolve this with specific choices of frame (equivalently, ribbons) along the curve. The present work takes an approach that is different in spirit -- the issue is resolved by separating out the intervals on which the curvature is nonzero, and then connecting frames for those intervals by jumping along the parts where the curvature is zero.

This work is creative, interesting and a signifigant contribution.

This report concludes with two extremely minor suggestions for changes in the final published form:

In the main theorem starting at the bottom of page 7:

1) the first line has a misspelling
"arclengh" ---> "arclength"

2) the third line refers to a "script K", and perhaps it should be mentioned within the theorem that this "script K" was defined in equation (2.5)

Publication of this submission is fully recommended.

Decision letter (RSPA-2020-0617.R0)

22-Dec-2020

Dear Dr Seguin,

On behalf of the Editor, I am pleased to inform you that your Manuscript RSPA-2020-0617 entitled "Bridging the gap between rectifying developables and tangent developables: a family of developable surfaces associated with a space curve" has been accepted for publication subject to minor revisions in Proceedings A. Please find the referees' comments below.

The reviewer(s) have recommended publication, but also suggest some minor revisions to your manuscript. Therefore, I invite you to respond to the reviewer(s)' comments and revise your manuscript. Please note that we have a strict upper limit of 28 pages for each paper. Please endeavour to incorporate any revisions while keeping the paper within journal limits. Please note that page charges are made on all papers longer than 20 pages. If you cannot pay these charges you must reduce your paper to 20 pages before submitting your revision. Your paper has been ESTIMATED to be 13 pages. We cannot proceed with typesetting your paper without your agreement to meet page charges in full should the paper exceed 20 pages when typeset. If you have any questions, please do get in touch.

It is a condition of publication that you submit the revised version of your manuscript by 4th January 2021. If you do not think you will be able to meet this date please let me know in advance of the due date.

To revise your manuscript, log into https://mc.manuscriptcentral.com/prsa and enter your Author Centre, where you will find your manuscript title listed under "Manuscripts with Decisions." Under "Actions," click on "Create a Revision." Your manuscript number has been appended to denote a revision.

You will be unable to make your revisions on the originally submitted version of the manuscript. Instead, revise your manuscript and upload a new version through your Author Centre.

When submitting your revised manuscript, you will be able to respond to the comments made by the referee(s) and upload a file "Response to Referees" in Step 1: "View and Respond to Decision Letter". You can use this to document any changes you make to the original manuscript. In order to expedite the processing of the revised manuscript, please be as specific as possible in your response to the referee(s).

IMPORTANT: Your original files are available to you when you upload your revised manuscript. Please delete any redundant files before completing the submission process.

In addition to addressing all of the reviewers' and editor's comments, your revised manuscript MUST contain the following sections before the reference list (for any heading that does not apply to your work, please include a comment to this effect):

• Acknowledgements
• Funding statement

See https://royalsociety.org/journals/authors/author-guidelines/ for further details.

When uploading your revised files, please make sure that you include the following as we cannot proceed without these:

1) A text file of the manuscript (doc, txt, rtf or tex), including the references, tables (including captions) and figure captions. Please remove any tracked changes from the text before submission. PDF files are not an accepted format for the "Main Document".

2) A separate electronic file of each figure (tif, eps or print-quality pdf preferred). The format should be produced directly from original creation package, or original software format.

3) Electronic Supplementary Material (ESM): all supplementary materials accompanying an accepted article will be treated as in their final form. Note that the Royal Society will not edit or typeset supplementary material and it will be hosted as provided. Please ensure that the supplementary material includes the paper details where possible (authors, article title, journal name). Supplementary files will be published alongside the paper on the journal website and posted on the online figshare repository (https://figshare.com). The heading and legend provided for each supplementary file during the submission process will be used to create the figshare page, so please ensure these are accurate and informative so that your files can be found in searches. Files on figshare will be made available approximately one week before the accompanying article so that the supplementary material can be attributed a unique DOI.
Alternatively you may upload a zip folder containing all source files for your manuscript as described above with a PDF as your "Main Document". This should be the full paper as it appears when compiled from the individual files supplied in the zip folder.

Article Funder

Please ensure you fill in the Article Funder question on page 2 to ensure the correct data is collected for FundRef (http://www.crossref.org/fundref/).

Media summary

Please ensure you include a short non-technical summary (up to 100 words) of the key findings/importance of your paper. This will be used for to promote your work and marketing purposes (e.g. press releases). The summary should be prepared using the following guidelines:

*Write simple English: this is intended for the general public. Please explain any essential technical terms in a short and simple manner.
*Describe (a) the study (b) its key findings and (c) its implications.
*State why this work is newsworthy, be concise and do not overstate (true 'breakthroughs' are a rarity).
*Ensure that you include valid contact details for the lead author (institutional address, email address, telephone number).

Cover images

We welcome submissions of images for possible use on the cover of Proceedings A. Images should be square in dimension and please ensure that you obtain all relevant copyright permissions before submitting the image to us. If you would like to submit an image for consideration please send your image to proceedingsa@royalsociety.org

Once again, thank you for submitting your manuscript to Proceedings A and I look forward to receiving your revision. If you have any questions at all, please do not hesitate to get in touch.

Best wishes
Raminder Shergill
proceedingsa@royalsociety.org
Proceedings A

on behalf of
Professor Achim Kempf
Board Member
Proceedings A

Reviewer(s)' Comments to Author:

Referee: 1

Comments to the Author(s)
From a space curve, there are two familiar constructions of a developable surface. The tangent developable whose rulings are tangent to the curve at each point and relative to the surface the absolute value of geodesic curvature of the curve is equal to the curvature of the curve. The other construction is the rectifying developable. The geodesic curvature of the curve relative to this surface vanishes. The authors show that there is a family of developable surfaces that can be generated from a curve, one surface for each function k that is defined on the curve and satisfies |k|<κ, and that the geodesic curvature of the curve relative to each such constructed surface satifies k_g≤k. The theoretical background is interesting. If the authors explain the equations (2.12) and (2.13) in detail or explain them by pictures, I think it will be more acceptable.

Referee: 2

Comments to the Author(s)
I recommend the paper "Bridging the gap between rectifying developables and tangent developables: a family of developable surfaces associated with a space curve" by S. Brian, Y.-C. Chen and E. Fried for publication after considering my comments below.

---Please consider to include two other very recent papers on developable surfaces:

O. Stein, E. Grinspun and K. Crane:
Developability of Triangle Meshes.
ACM Trans. Graph, 37(4), 2018.

M. Rabinovich, T. Hoffmann and O. Sorkine-Hornung:
The Shape Space of Discrete Orthogonal Geodesic Nets,
ACM Trans. Graph, 37(6), 2018.

---The authors use the notion "pointwise vanishing ..." throughout the paper. I would appreciate if you could rephrase it. For example, I would say that minimal surfaces have vanishing mean curvature and I would not say that minimal surfaces have *pointwise* vanishing mean curvature.

---In "classical" differential geometry, a curve, parametrized by d, is *regular* iff d' is not vanishing. However, the authors introduce the notion of "piecewise regular" (line 40) by d'' = 0 only on a finite number of disjoint subintervals. That is a bit confusing notion. Can you change that?

---line 49: I think you are talking about a ruled surface with vanishing mean curvature. However, such a surface does not necessarily have vanishing Gaussian curvature as the helicoid is a counterexample. And therefore, S is not necessarily flat. Please reconsider that statement.

---Why does Struik (1961) not get a number in the Reference section?

Referee: 3

Comments to the Author(s)

This is a positive review of the article entitled

Bridging the gap between rectifying developables and
tangent developables: a family of developable surfaces
associated with a space curve

written by Seguin, Chen and Fried.

Publication in Proc. Royal Soc. A is recommended withoutreservation.

This submission demonstrates the existence of a continuum of developables built from a single space curve, including the tangent developable and rectifying developable as outliers within the collection. It appears to be an infinite dimensional family, as it is parametrized by a family of functions.

There is a thorough explanation of historical context and applications, which indicates why the authors are interested in fixing the initial curve for constructing the surfaces, and why the curve is allowed to have intervals of zero curvature, and why the surfaces are not assumed to be C-infinity smooth.

Ambiguity for the Frenet frame results at points where the initial curve has curvature zero. Mathematicians generally resolve this with specific choices of frame (equivalently, ribbons) along the curve. The present work takes an approach that is different in spirit -- the issue is resolved by separating out the intervals on which the curvature is nonzero, and then connecting frames for those intervals by jumping along the parts where the curvature is zero.

This work is creative, interesting and a signifigant contribution.

This report concludes with two extremely minor suggestions for changes in the final published form:

In the main theorem starting at the bottom of page 7:

1) the first line has a misspelling
"arclengh" ---> "arclength"

2) the third line refers to a "script K", and perhaps it should be mentioned within the theorem that this "script K" was defined in equation (2.5)

Publication of this submission is fully recommended.

Board member pre-assessment comments (if available):

Board Member: 1
Comments to Author(s):
This paper should be acceptable after the referees' comments have been suitable addressed.

Decision letter (RSPA-2020-0617.R1)

13-Jan-2021

Dear Dr Seguin

I am pleased to inform you that your manuscript entitled "Bridging the gap between rectifying developables and tangent developables: a family of developable surfaces associated with a space curve" has been accepted in its final form for publication in Proceedings A.

Our Production Office will be in contact with you in due course. You can expect to receive a proof of your article soon. Please contact the office to let us know if you are likely to be away from e-mail in the near future. If you do not notify us and comments are not received within 5 days of sending the proof, we may publish the paper as it stands.

Open access
You are invited to opt for open access, our author pays publishing model. Payment of open access fees will enable your article to be made freely available via the Royal Society website as soon as it is ready for publication. For more information about open access please visit https://royalsociety.org/journals/authors/which-journal/open-access/. The open access fee for this journal is £1700/$2380/€2040 per article. VAT will be charged where applicable.

Note that if you have opted for open access then payment will be required before the article is published – payment instructions will follow shortly.

If you wish to opt for open access then please inform the editorial office (proceedingsa@royalsociety.org) as soon as possible.

Your article has been estimated as being 14 pages long. Our Production Office will inform you of the exact length at the proof stage.

Proceedings A levies charges for articles which exceed 20 printed pages. (based upon approximately 540 words or 2 figures per page). Articles exceeding this limit will incur page charges of £150 per page or part page, plus VAT (where applicable).

Under the terms of our licence to publish you may post the author generated postprint (ie. your accepted version not the final typeset version) of your manuscript at any time and this can be made freely available. Postprints can be deposited on a personal or institutional website, or a recognised server/repository. Please note however, that the reporting of postprints is subject to a media embargo, and that the status the manuscript should be made clear. Upon publication of the definitive version on the publisher's site, full details and a link should be added.

You can cite the article in advance of publication using its DOI. The DOI will take the form: 10.1098/rspa.XXXX.YYYY, where XXXX and YYYY are the last 8 digits of your manuscript number (eg. if your manuscript number is RSPA-2017-1234 the DOI would be 10.1098/rspa.2017.1234).

For tips on promoting your accepted paper see our blog post: https://royalsociety.org/blog/2020/07/promoting-your-latest-paper-and-tracking-your-results/

On behalf of the Editor of Proceedings A, we look forward to your continued contributions to the Journal.

Sincerely,
Raminder Shergill
proceedingsa@royalsociety.org
